# Monitoring of Enoxaparin during Hemodialysis Covered by Regional Citrate Anticoagulation in Acute Kidney Injury: A Prospective Cohort Study

**DOI:** 10.3390/jcm10194491

**Published:** 2021-09-29

**Authors:** Marion Wiegele, Dieter Adelmann, Christoph Dibiasi, Andrè Pausch, Andreas Baierl, Eva Schaden

**Affiliations:** 1 Department of Anesthesia, Critical Care and Pain Medicine, Division of General Anesthesia and Intensive Care Medicine, Medical University of Vienna, 1090 Vienna, Austria; marion.wiegele@meduniwien.ac.at (M.W.); christoph.dibiasi@meduniwien.ac.at (C.D.); andre.pausch@meduniwien.ac.at (A.P.); 2 Department of Anesthesia & Perioperative Care, University of California, San Francisco, CA 94143, USA; dieter.adelmann@ucsf.edu; 3 Ludwig Boltzmann Institute for Digital Health and Patient Safety, Medical University of Vienna, 1090 Vienna, Austria; 4 Department of Statistic and Operations Research, University of Vienna, 1090 Vienna, Austria; andreas.baierl@univie.ac.at

**Keywords:** acute kidney injury, anti-Xa, drug monitoring, enoxaparin, renal replacement therapy, rotational thromboelastometry

## Abstract

Background: Current guidelines recommend the monitoring of anti-factor Xa (anti-Xa) levels to avoid an accumulation of low-molecular-weight heparins in patients with acute kidney injury, but there is no evidence on how to proceed with such monitoring during continuous renal replacement therapy. Against this background, we investigated the potential accumulation of enoxaparin administered subcutaneously for venous thromboembolism prophylaxis in critically ill patients during continuous renal replacement therapy covered by regional citrate anticoagulation. Methods: Anti-Xa levels were measured at baseline (≤12 h before renal replacement therapy) and on three consecutive days (A to C) when enoxaparin had reached trough levels. Supplementary testing included modified assays of rotational thromboelastometry known to be highly sensitive for low-molecular-weight heparins. Results: The 16 men and 13 women included were adults comparable in age, body mass index, thromboembolism risk assessment, and clinical severity of the disease. Throughout the four examinations, the median trough levels of anti-Xa remained below the detection limit of the test (<0.1 IU mL^−1^), with interquartile ranges of <0.1 to 0.14 IU mL^−1^ at baseline and <0.1 to 0.16 IU mL^−1^ on days A/B/C. All rotational thromboelastometry parameters of clot initiation and clot formation dynamics did not significantly change from baseline to day C. Conclusions: Neither anti-Xa levels nor modified assays of rotational thromboelastometry revealed any accumulation of enoxaparin administered for thromboprophylaxis during continuous renal replacement therapy covered by regional citrate anticoagulation. Although generally recommended in patients with acute kidney injury, monitoring of anti-Xa levels should be questioned in this defined setting.

## 1. Introduction

The delicate hemostatic balance of critically ill patients has been extensively discussed ever since the mid-2000s [1,2]. Depending on underlying etiologies and therapeutic interventions, patients may present with a hypercoagulable state carrying an increased risk of venous thromboembolism, alternatively be at risk of bleeding, or have both risks at the same time. The latter is particularly true in perioperative settings, as tissue trauma induces procoagulant hemostatic alterations while bleeding prevention is a major requirement after surgery [3,4]. This conflict of risks is compounded in situations of renal failure and continuous renal replacement therapy (CRRT) [5].

As procoagulatory and proinflammatory processes are activated by extracorporeal circulation, thus adding to the risk of thromboembolic events and notably of filter clotting, anticoagulation is recommended during CRRT [5]. Effective protection of this extracorporeal circuit by regional citrate anticoagulation does not obviate the need for venous thromboembolism prophylaxis [5,6]. Prophylaxis by systemic administration of a low-molecular-weight heparin (LMWH) as recommended by current guidelines for critically ill patients raises the question of how to avoid an accumulation of this drug [7,8].

In patients with severe kidney injury, it has been suggested to deal with this concern of LMWH accumulation via monitoring of anti-factor Xa (anti-Xa) trough levels [9]. Apart from that, no evidence is available on how to proceed with such monitoring in patients receiving LMWH thromboprophylaxis during CRRT with regional citrate anticoagulation.

To address the question of LMWH accumulation in critically ill patients, we designed a prospective clinical investigation into the development of plasma anti-Xa levels over the course of CRRT with regional citrate anticoagulation, supplemented by modified and highly LMWH-sensitive assays of rotational thromboelastometry (ROTEM) to uncover minor changes below the detection limit of anti-Xa testing.

## 2. Materials and Methods

This prospective observational trial was conducted in accordance with the 1964 Declaration of Helsinki and its later amendments. It was approved by the ethics committee (institutional review board) of Medical University of Vienna on 4 July 2012 (ref. 1416/2012) and was registered at the German Clinical Trials Register (https://www.drks.de, accessed on 29 August 2012) (DRKS00004336). All patients gave informed consent to participate after having received comprehensive information about the nature and scope of the study and the examinations to be performed. None of them received a stipend, and all agreed to their anonymized data being published. Results of thrombin generation and platelet function tests in some of the patients have recently been published [10].

### 2.1. Eligible Patients and Exclusion Criteria

Adult patients (>18 years) with acute kidney injury indicating CRRT were prospectively screened for eligibility at three surgical Intensive Care Units (ICUs) at Medical University of Vienna, Austria, between 7 February 2013 and 21 November 2018. All of these were managed by continuous veno-venous hemodialysis (CVVHD) with regional citrate anticoagulation using commercially available equipment, materials, solutions (multiFiltrate Ci-Ca^®^, Ultraflux AV 1000s, Ci-Ca dialysat K2, sodium citrate 4%, 0.5 M CaCl_2_; Fresenius Medical Care, Hof an der Saale, Germany), and default initial settings for adults (blood flow/effluent flow ratio: 1/20; calcium: 1.7 mmol L^−1^; citrate: 4 mmol L^−1^) followed by a standardized protocol of adjusting flow rates, as dictated by metabolic disturbances, reported in detail elsewhere [11]. In accordance with current guidelines, venous thromboembolism prophylaxis was provided by the subcutaneous administration of enoxaparin 4000 IU once daily and via intermittent pneumatic compression [7]. Patients with impaired hemostasis (due to conditions such as known coagulation disorders, therapeutic anticoagulation, major bleeding, or severe liver dysfunction) at the outset of CRRT or previously were excluded.

### 2.2. Parameters Collected for Evaluation

Based on blood samples drawn from indwelling arterial or central venous catheters, coagulation assays were performed at baseline (≤12 h before the start of CRRT) and on three consecutive days (recommended service life of the hemofilter) when the enoxaparin reached trough levels 12 to 24 h after administration. Patient data were extracted from two automated patient data management systems: CareVue (Agilent Technologies, Santa Clara, CA, USA) and, launched in April 2013, IntelliSpace Critical Care and Anesthesia (ICCA; Philips Healthcare, Vienna, Austria). They included Caprini scores for thromboembolism risk assessment [12], medical histories, and routinely collected details on medications (including vasopressors), fluid balance, parameters of renal function, transfusion requirements, and the use of coagulation factor concentrates.

### 2.3. Laboratory Assessments and Reference Ranges (RR)

Conventional coagulation and rotational thromboelastometry (ROTEM^®^ delta; TEM Innovations, Munich, Germany) assays were performed—the latter by anesthetists trained in point-of-care diagnostics (M.W.; D.A.)—from citrated (trisodium citrate 3.8% 9:1 *v*/*v*) plasma in appropriate tubes (Vacuette^®^; Greiner Bio-One, Kremsmünster, Austria). Parameters assessed by the conventional assays were prothrombin time using the Owren method (PT; RR: 70−125%; SI conversion factor: 0.01), activated partial thromboplastin time (aPTT; RR: 27–41 s), antithrombin (AT; RR: 80–120%; SI conversion factor: 0.01), and fibrinogen using the Clauss method (RR: 200–400 mg dL^−1^; SI conversion factor: 0.01). The same coagulometer (STA R Max 2^®^; Diagnostica Stago SAS, Asnières-sur-Seine, France) was employed to assess anti-Xa levels (RR: < 0.1 IU mL^−1^) using a proprietary test (STA^®^-Liquid Anti-Xa 00311 and 00322; Diagnostica Stago SAS, Asnières-sur-Seine, France). Platelet counts (RR: 150–350 G L^−1^) were determined from EDTA tubes (Vacuette^®^; Greiner Bio-One, Kremsmünster, Austria) with an automated analyzer (XE-2100; Sysmex, Kobe, Japan).

The ROTEM investigations included a regular extrinsically activated (EXTEM) assay, which was conducted as per the manufacturer’s instructions to detect extrinsically activated clotting dynamics [13,14]. In addition, we used two modified assays known to correlate with anti-Xa levels in vitro: low-tissue-factor (low-TF) and prothrombinase-induced clotting time (PiCT) ROTEM [15]. EXTEM parameters included coagulation time (CT, RR: 38–79 s) and clot formation time (CFT, RR: 34–159 s) as the main indicators of clot initiation and polymerization, maximum clot firmness (MCF, RR: 50–72 mm) reflecting clot stability, and maximum velocity (maxV, mm s^−1^) as well as time until maximum velocity (maxVt, s) of clot formation to identify the dynamic properties of coagulation and to quantify thrombin generation [16].

For the PiCT assay, 20 µL of reagent (Pefakit^®^; Pentapharm, Munich, Germany) containing Xa coagulation factor and RVV-V (a factor-5 activator derived from viper venom) was reconstituted and dispensed into 300 µL of whole blood, adding 20 µL of CaCl_2_ to initiate clotting after 3 min of incubation. For the low-TF assay, 100 µL of reconstituted lyophilized recombinant tissue factor (Dade Innovin^®^; Siemens Healthcare Diagnostics Products, Marburg, Germany) was diluted with 100 mL of distilled water and 20 µL of the low-TF reagent dispensed along with 20 µL of CaCl_2_ into a cup containing 300 µL of whole blood (TF concentration in the final test sample: 1:1000) to initiate clotting. No reference ranges are available for the two modified ROTEM applications.

### 2.4. Diagnosis of Venous Thromboembolism

A compression ultrasound of the lower extremities was performed, for study purposes only, to rule out deep vein thrombosis (DVT) prior to inclusion in the study, on day C, and at discharge from the ICU. This did not include screening for distal or superficial vein thrombosis, but any lack of compressibility on the B-mode ultrasound of either the common femoral vein or the venous system down to the popliteal vein was considered diagnostic for proximal deep vein thrombosis. Central or lobar (but not subsegmental) pulmonary embolism was included if confirmed by pulmonary angiography.

### 2.5. Statistical Analysis

Median values are used to report the central tendencies of continuous variables, and their dispersion is indicated by the first and third quartiles. Basic data were evaluated based on sex, given the inherently increased risk of thromboembolic events in men [17]. Mann–Whitney U tests to identify any differences in the demographic continuous variables between the sexes were followed by Wilcoxon signed rank tests for paired samples to assess the measurements of all continuous variables for differences between baseline and day C. In addition, scatterplots with simple linear regressions were obtained to illustrate how anti-Xa levels were associated with clotting time. All tests were two-sided, with *p*-values < 0.05 considered statistically significant, and all statistical analyses were performed with R statistical software (v. 4.02; R Core Team 2020, R Foundation for Statistical Computing, Vienna, Austria) [18].

## 3. Results

### 3.1. Patient Characteristics

Over 4900 patients were screened for eligibility during the six-year prospective timeframe. A total of 16 male and 13 female patients (*n* = 29) could be included for evaluation. None of them developed pulmonary embolism or deep vein thrombosis while in the ICU. As is apparent from Table 1, no significant differences between the sexes were noted regarding age, body mass index, thromboembolism risk assessment, or clinical severity of the disease.

Table 2 lists the findings for renal function and conventional coagulation parameters, as well as for procoagulant medication and blood transfusion requirements at baseline and during each of the three consecutive ICU days. Only three patients required transfusion of >2 units of packed red blood cells (PRBC) indicative of major bleeding complications. All three of them received LMWH therapy on the same day since anti-Xa trough levels remained below 0.1 IU mL^−1^. ATIII was substituted in a total of three patients: prior to the start of citrate dialysis (baseline) and at day A in one patient. A single shot of ATIII was administered in two patients, once at baseline and once at day A of the study. The increase in ATIII plasma levels we found over time was not significant and most likely resulted from the intended fluid removal during CRRT, which might have led to a concomitant “pseudo”-increase in plasma concentrations.

### 3.2. Anti-Xa Levels during CRRT

**Figure 1:**
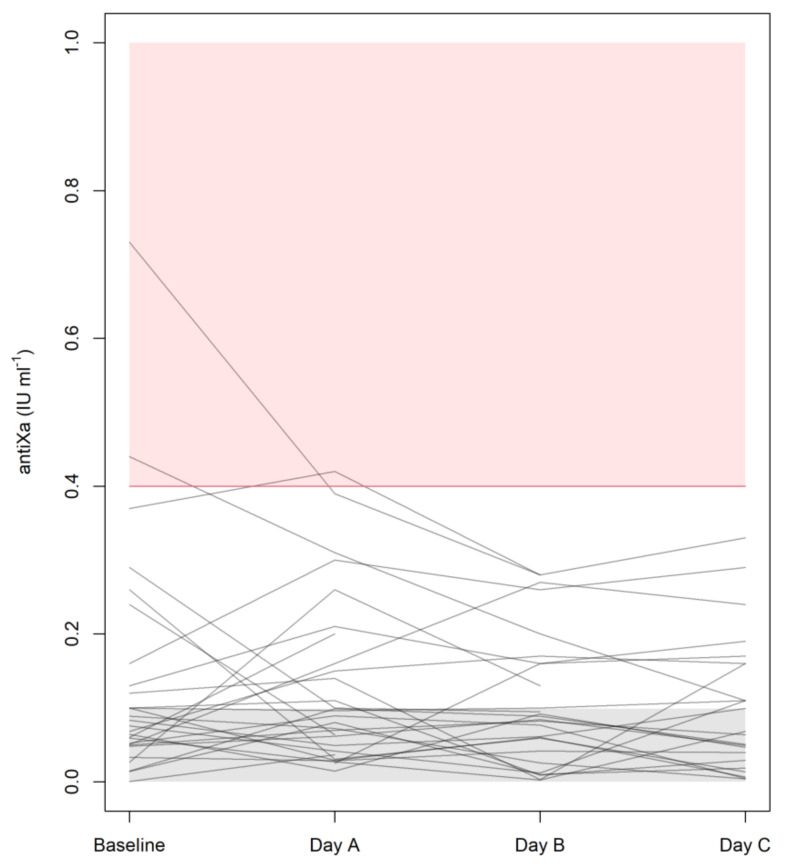
Plasma anti-Xa levels at baseline and during each day in the ICU. Target levels for (semi)therapeutic anticoagulation are indicated by the pink zone, whereas the gray zone at the bottom has been populated with random values down to zero to illustrate results below the detection limit of the test (<0.1 IU mL^−1^).

Figure 1 illustrates how the plasma trough levels of anti-Xa developed over the course of these four examinations. It is important to note that all median trough levels did remain below the detection limit (<0.1 IU mL^−1^) of the test, with interquartile ranges of <0.1 to 0.14 IU mL^−1^ at baseline and <0.1 to 0.16 IU mL^−1^ on days A, B, and C. To put this consistent finding of mean trough levels <0.1 IU mL^−1^ into perspective, Figure 1 also makes it clear that some measurable values ranging from 0.2 to 0.4 IU mL^−1^ were seen in individual patients. Only one patient, however, exhibited one mean trough level of anti-Xa amounting to >0.4 IU mL^−1^, and this singular finding on day A was also preceded by a rather high value at baseline and was followed by a considerable decrease on day B. LMWH therapy was withheld in three patients on day A (one presenting with an anti-Xa level of 0.31 IU mL^−1^) and in two patients on day B (anti-Xa: 0.20 or 0.28 IU mL^−1^).

### 3.3. ROTEM Parameters during CRRT

**Figure 2:**
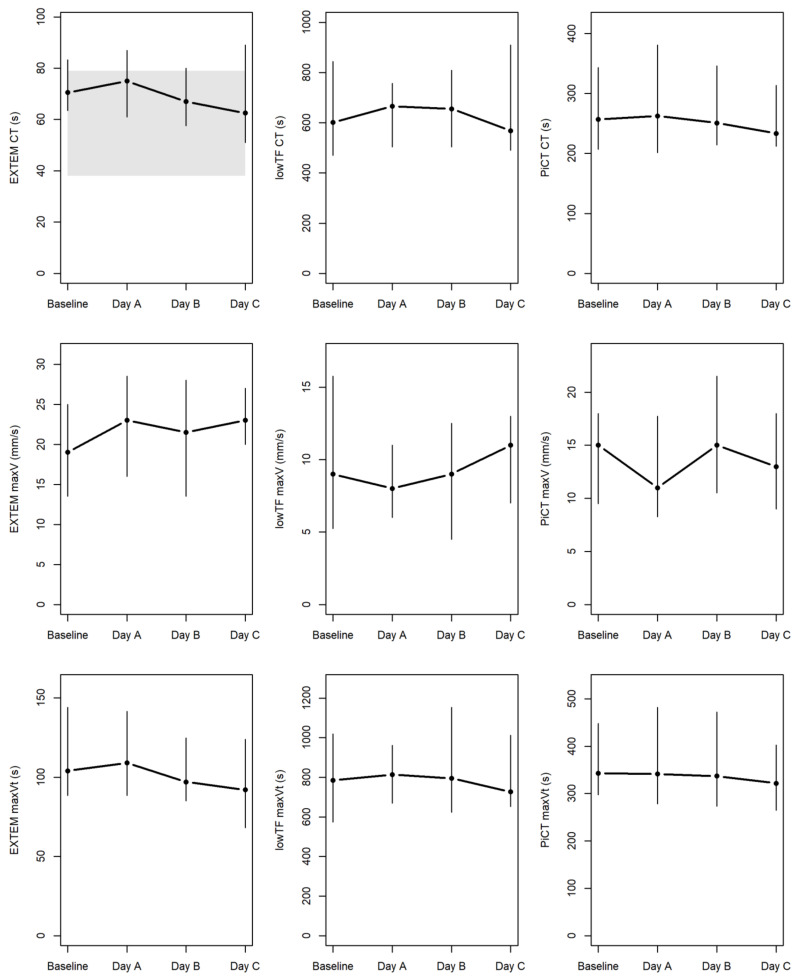
ROTEM data at baseline and during each day in the ICU. Parameters of clotting time (CT), maximum velocity (maxV), and time to maximum velocity (maxVt) were measured by standard EXTEM (i.e., extrinsically activated ROTEM) and by two modified assays of low-TF (low-tissue-factor) ROTEM and PiCT (prothrombinase-induced clotting) NATEM (i.e., non-activated ROTEM) known to correlate greatly with anti-Xa levels [14]. Median values for the four examinations are connected by horizontal lines, with vertical lines extending from the 1st to 3rd quartiles. The area highlighted in gray indicates the only available reference range (for CT by EXTEM).

**Figure A1:**
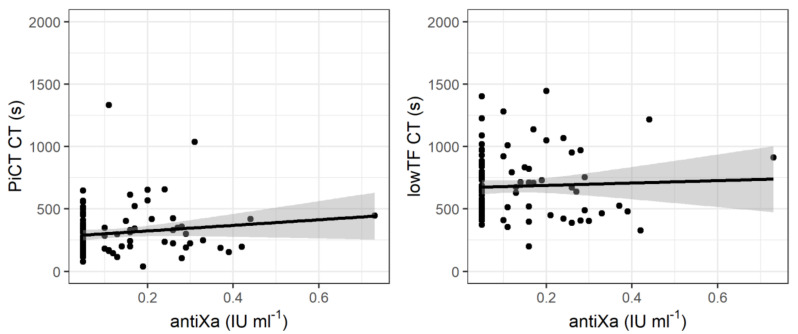
CT, maxV, and maxVt in the regular EXTEM and the two modified (PiCT and low-TF) assays. PiCT: prothrombinase-induced clotting; low-TF: low-tissue-factor; CT: clotting time; maxV: maximum velocity; maxVt: time to maximum velocity; EXTEM: extrinsically activated rotational thrombelastometry assay.

Table 3 summarizes the results of the various standard and modified ROTEM assays. No significant differences were found on day C of hemodialysis compared to the baseline for any of the parameters pertaining to clot initiation, clot formation dynamics (clot formation time, maximum velocity, and time to maximum velocity), and maximum clot firmness. Figure A1 within the Appendix illustrates the developments of CT, maxV, and maxVt in the regular EXTEM and the two modified (PiCT and low-TF) assays. The linear regressions in Figure 2 revealed that clot initiation did not correlate with anti-Xa trough levels in the modified PiCT (rho = 0.11; *p* = 0.29) and low-TF (rho = 0.01; *p* = 0.88) assays throughout the four examinations at baseline and on days A to C of hemodialysis.

## 4. Discussion

Our anti-Xa and modified ROTEM assays performed at baseline and over three days demonstrated that venous thromboembolism prophylaxis with subcutaneous enoxaparin did not lead to any noteworthy accumulation of LMWH during CRRT with citrate anticoagulation in surgical ICU patients with acute kidney injury. The finding that median anti-Xa trough levels remained below the detection limit on each of three consecutive CRRT days was also confirmed by observing no significant differences in ROTEM parameters of clot initiation, clot formation dynamics, and clot firmness between baseline and day C.

Patients managed by CRRT in the ICU, even when regional citrate anticoagulation of the extracorporeal circuit is provided as the recommended standard of care, still require venous thromboembolism prophylaxis [5,6]. Recommendations exist that anti-Xa trough levels should be monitored during LMWH anticoagulation of patients with acute kidney injury, such that the dose can be reduced as needed to prevent accumulation of the drug. Nevertheless, it has been unclear how to approach acute cases treated by CRRT.

Over 15 years have passed since, after determining sieving and saturation coefficients of enoxaparin from dialysate/ultrafiltrate and plasma samples, and finding that enoxaparin passed through polysulfone membranes, Isla et al. [19] called for more research into dose adjustments during CRRT. To our knowledge, the present article reports on the first study dealing with the dynamics of plasma anti-Xa levels in ICU patients who received both regional citrate anticoagulation and systemic LMWH treatment as recommended for venous thromboembolism prophylaxis [7].

There is an ongoing discussion about the usefulness of anti-Xa determination to guide anticoagulant therapy in critically ill patients receiving an LMWH. To some degree, this inconclusiveness may be due to the heterogeneity of the ICU population or imprecise clinical endpoints. However, the use of anti-Xa raises a few questions in and by itself, given the uncertainties about whether target or trough [8,20,21,22] and exactly what plasma [23,24] levels should be achieved. The most notable point of debate concerns the reliability of a drug concentration marker as a surrogate marker of clinical implications [8,25].

With regard to therapeutic anticoagulation, precise recommendations for monitoring LMWH concentrations and arriving at anti-Xa target levels of 0.6–1.0 IU mL^−1^ are available [26]. Whether anti-Xa is also suitable to guide LMWH application for thromboembolism prophylaxis is a different question, as anti-Xa levels reflecting adequate prophylaxis have yet to be defined [8,27] and monitoring to avoid an accumulation of the drug is not generally required unless in high-risk patients (e.g., those with severe renal injury defined by a creatinine clearance <30 mL min^−1^) [7,26]. Current recommendations are to determine the trough levels of anti-Xa in this situation [8,28]. Our finding of the trough levels revealing no accumulation of an LMWH administered for thromboembolism prophylaxis seems to obviate the need for LMWH monitoring during CRRT.

The manufacturer of the LMWH-calibrated anti-Xa assay used in this study has defined a lower detection limit of < 0.1 IU mL^−1^. In the absence of more detailed information below this value, and considering the potentially increased excretion of enoxaparin [19], we performed two modified ROTEM assays, which had been shown in vitro to strongly correlate with plasma LMWH levels and prolonged clot initiation [15,29], to uncover minor changes underneath this detection threshold of anti-Xa. Indeed, both of these PiCT and low-TF tests had been found to reveal linear increases in clotting time (CT) with increasing LMWH concentrations starting from 0.05 IU mL^−1^ [15]. Based on these previous findings, we investigated the parameters of clot initiation (CT) and clot formation dynamics (CFT, maxV, and maxVt) using a conventional EXTEM assay and both of these modified PiCT and low-TF assays. No significant differences were found in any of the parameters between baseline and day C of the CRRT procedures in the ICU.

Despite the previous in vitro studies [15,29], our finding of no significant correlations between clotting times (CT) and plasma anti-Xa levels in both the PiCT (*p* = 0.147) and the low-TF (*p* = 0.152) modified ROTEM assays (see Figure 2) might be due to <0.1 IU mL^−1^ being a rather ‘broad’ definition of the lower detection limit. On closer inspection, the CT values of 234–257 s were comparable to the ≈200–250 s reported previously for anti-Xa levels at 0.05–0.1 IU mL^−1^ [15]. While we did not find these modified tests to correlate with anti-Xa plasma levels, we did observe prolonged clotting times in both of them, with patient-based values in the low-TF modified ROTEM assay almost twice as long as previously noted in healthy volunteers [15]. This finding does suggest that an effect of the LMWH may be present despite anti-Xa levels being below the detection limit.

Possible limitations of the present study, in addition to its small sample size, may have included heterogeneity of the cohort, even though all patients were recruited at surgical ICUs and were shown to be comparable with regard to clinical severity, demographic characteristics, and anticoagulant therapies. Fibrinogen levels, indicating activation of the hemostatic system, remained stable without significant alterations from baseline until day C. Given our aim to track the development of plasma anti-Xa over time, we did not determine its levels in the ultrafiltrate, and the question of enoxaparin passing through CRRT-related membranes has been addressed before [19,30]. It is, however, fair to add that not all results of this study may hold up to scrutiny given the use of different membranes and default flow rates for continuous veno-venous hemodialysis. Lastly, LMWH therapy was discontinued in five patients on day A (*n* = 3) or B (*n* = 2) due to high anti-Xa levels, although none of these five patients presented with bleeding complications, emphasizing once again the questionable utility of anti-Xa as a surrogate marker of clinical implications.

## 5. Conclusions

Monitoring of anti-Xa levels did not reveal any accumulation of enoxaparin after systemic administration for thromboprophylaxis during CRRT covered by regional citrate anticoagulation. Hence, such monitoring, which is generally recommended in patients with acute kidney injury, should be questioned in this defined setting.

## Figures and Tables

**Table 1:** Pertinent patient data broken down by sex.

	N	Median	Male	Female	*p* Value
Sex	n/a	n/a	16	13	n/a
Age (years)	29	66 (58; 72)	67 (63; 72)	65 (56; 73)	0.83
BMI	29	25.7 (24.4; 29)	25 (24.1; 27.9)	28.4 (25; 31.1)	0.23
SAPS III	29	65 (59.5; 80)	62 (55; 72)	71 (62; 80)	0.16
Caprini score	29	8 (6; 9)	9 (6; 9)	8 (6; 9)	0.4

Data are presented as median values with 1st and 3rd quartiles. SAPS III (simplified acute physiology score) values are severity rating of disease, Caprini scores represent the findings of risk assessment for venous thromboembolism, and comparability between the sexes is indicated by the *p*-values (Mann–Whitney U testing). BMI: body mass index.

**Table 2:** Laboratory parameters, procoagulant medication, and blood transfusion requirements.

	*N*	Baseline	Day A	Day B	Day C	*p*
**Renal function, fluid balance, vasopressor therapy**						
BUN (mg dL^−1^)	27	74.0 (37.1; 99.6)	42.0 (28.1; 61.1)	26.2 (22.1; 37.0)	22.8 (18.6; 29.8)	<0.001
Creatinine (mg dL^−1^)	27	3.09 (2.36; 4.50)	2.01 (1.76; 2.69)	1.40 (1.22; 1.95)	1.36 (1.02; 2.03)	<0.001
Urine output (mL day^−1^)	25	630 (275; 1043)	380 (230; 750)	220 (59; 525)	150 (0; 410)	<0.001
Blood flow rates, CVVHD (mL min^−1^)	29	n/a	100 (100;100)	100 (100; 100)	100 (100;100)	n/a
Fluid balance (mL day^−1^)	24	1560 (217; 2896)	690 (−522; 2456)	7 (−392; 659)	−326 (−1326; 383)	0.16
Norepinephrine (*n*)	29	15	14	10	7	n/a
Norepinephrine (mcg kg^−1^ min^−1^)	29	0.003 (0; 0.23)	0 (0; 0.08)	0 (0; 0.05)	0 (0; 0.05)	n/a
**Conventional coagulation assays and platelet counts**						
PT Owren (%)	27	62 (47; 86)	70 (50;87)	73 (55; 91)	79 (63; 92)	0.14
aPTT (s)	27	39.3 (36.9; 47.9)	41.9 (38.4; 46.4)	40.2 (37.9; 46.8)	38.6 (35.1; 43)	0.46
Fibrinogen (mg dL^−1^)	27	509 (390; 652)	558 (378; 689)	574 (416; 673)	579 (454; 650)	0.95
AT III (%)	26	64 (45; 97)	68 (50; 105)	71 (58; 93)	86 (63; 98)	0.06
Platetet count (G L^−1^)	27	183 (106; 230)	180 (108; 222)	161 (109; 196)	142 (123; 193)	0.12
**Procoagulant drugs (number of patients receiving ≥ 1 application)**						
Tranexamic acid	1	1	0	0	0	n/a
Fibrinogen concentrate	2	1	1	0	0	n/a
Prothrombin complex concentrate	1	1	0	0	0	n/a
AT III concentrate	2	2	0	0	0	n/a
**Blood products (number of patients receiving > 2 units)**						
Packed red blood cells (PRBC)	3	3	1	0	0	n/a
Platelet concentrates	0	0	0	0	0	n/a
Fresh frozen plasma	1	0	0	1	0	n/a

Data are presented as median values with 1st and 3rd quartiles or as number of patients. The *n*-values listed in the second column from the left indicate the number of patients for whom evaluable results were available throughout all four examinations. The *p*-values in the rightmost column were obtained by comparing day C to baseline (Wilcoxon signed-rank tests). BUN (blood urea nitrogen) has a reference range of 6–23 mg dL^−1^ (SI conversion factor 0.3571). Serum creatinine has a reference range of 0.70–1.20 mg dL^−1^ (SI conversion factor 88.4). Norepinephrine in mcg kg^−1^ min^−1^ refers to the dosage administered at the time of blood sample collection. PT: prothrombin time; aPTT: activated partial thromboplastin time; AT: antithrombin.

**Table 3:** Results of the various standard and modified rotational thromboelastometry assays.

	*N*	Baseline	Day A	Day B	Day C	*p* Value
**Standard assay of extrinsically activated thromboelastometry (EXTEM)**						
CT (s)	22	71 (64; 83)	75 (61; 87)	67 (58; 80)	63 (51; 89)	0.37
CFT (s)	22	68 (52; 116)	66 (47; 100)	62 (48; 98)	63 (52; 86)	0.59
MCF (mm)	22	74 (62; 77)	71 (63; 78)	74 (61; 79)	74 (67; 79)	0.74
maxV (mm s^−1^)	17	19 (14; 25)	23 (16; 29)	22 (14; 28)	23 (20; 27)	0.15
maxVt (s)	17	104 (89; 144)	109 (89; 142)	97 (85; 125)	92 (68; 124)	0.15
**Modified assay of low-tissue-factor thromboelastometry (low-TF ROTEM)**						
CT (s)	22	602 (471; 845)	667 (504; 758)	656 (504; 809)	568 (491; 910)	0.97
CFT (s)	22	199 (140; 345)	220 (174; 386)	238 (186; 381)	231 (158; 370)	0.63
MCF (mm)	20	61 (52; 71)	58 (54; 70)	64 (54; 75)	68 (57; 74)	0.32
maxV (mm s^−1^)	15	9 (5; 16)	8 (6; 11)	9 (5; 13)	11 (7; 13)	0.89
maxVt (s)	15	786 (575; 1019)	814 (669; 961)	795 (623; 1153)	727 (652; 1012)	0.9
**Modified assay of prothrombinase-induced clotting (PiCT)**						
CT (s)	22	257 (207; 343)	263 (201; 381)	251 (214; 346)	234 (212; 314)	0.63
CFT (s)	22	137 (85; 179)	130 (83; 249)	145 (103; 184)	152 (109; 227)	0.1
MCF (mm)	21	69 (58; 75)	69 (59; 72)	68 (57; 75)	70 (57; 74)	0.19
maxV (mm s^−1^)	16	15 (10; 18)	11 (8; 18)	15 (11; 22)	13 (9; 18)	0.19
maxVt (s)	16	343 (298; 448)	342 (279; 482)	337 (273; 473)	322 (265; 403)	0.98

Data are presented as median values with 1st and 3rd quartiles. The *n*-values listed in the second column from the left indicate the number of patients for whom evaluable results were available throughout all four examinations. The *p*-values in the rightmost column were obtained by comparing day C to baseline (Wilcoxon signed-rank tests). CT: clotting time; CFT: clot formation time; maxV: maximum velocity; maxVt: time to maximum velocity; MCF: maximum clot firmness.

## Data Availability

The data presented in this study are available on request from the corresponding author.
